# Manual of Clinical Diagnosis

**Published:** 1891-10

**Authors:** 


MANUAL OF CLINICAL DIAGNOSIS. By Dr. OTTO SEIFERT, Privatdocent in Würzburg, and Dr. FRIEDRICH MULLER, Assistant der II. Med. Klinik in Berlin. Translated from the fifth German edition, enlarged and revised, with the permission of the author, by WILLIAM BUCKINGHAM CANFIELD, A. M., M. D. (Berlin), Fellow of American Academy of Medicine, etc., etc., etc. Second English edition, revised and enlarged, with fifty illustrations and one colored plate. G. P. Putnam's Sons, New York, 27 W. 23d St.; London, 27 King William St., Strand. The Knickerbocker Press. 1890.

The fact that this little book has reached a fifth edition in the German, and a second in English, proves that it is at least popular. Nevertheless, popularity is no criterion of the true value of a book which is intended as an educational work. This book belongs to a class which should be utterly condemned by all men who are striving to give thorough instruction to their students. In the preface to the first edition the authors say: "In selecting and arranging this material, we have been led by the experience gained in *holding courses*, and we have also endeavored to consider the *practical needs of the student* and physician by noting only what is reliable, and omitting everything self-evident and of secondary importance." In other words, this book is intended as one to which a student may refer just before going up for examination, and cram his head full of a certain number of important facts relative to diseases, so that he can pass his examination well, even though he knows nothing of the true *clinical* picture of any given disease.

In this work on "*clinical* diagnosis," the "omission of everything self-evident and of secondary importance," is so complete, that in discussing the various diseases which are characterized by changes in the blood, namely anemia, chlorosis, leucocytosis, leukemia, pseudo-leukemia, and pernicious anemia, no mention whatever of any truly *clinical* symptoms is made, but merely the changes in the character and numbers of the blood corpuscles are noted.

The subject of typhoid fever is dismissed in a little over half a page. No mention is made of diarrhea, nose-bleed, right iliac tenderness and gurgling, nor is the possibility of hemorrhage from the bowels or of intestinal perforation even referred to. Yet this is called a manual of *clinical* diagnosis. It is unnecessary to make further references, as the book is made on the same plan throughout, a plan which gives a very superficial knowledge, and is worthy only of utter condemnation by all who have at heart the thorough education of medical students. DeL. R.

